# Gamma-glutamyl transpeptidase to platelet and gamma-glutamyl transpeptidase to lymphocyte ratio in a sample of Chinese Han population

**DOI:** 10.1186/s12876-022-02509-1

**Published:** 2022-10-31

**Authors:** Weijie Chen, Danmian Hong, Zeliang Chen, Xiaoqing Dai, Jing Cao, Min Yu, Liping Li

**Affiliations:** 1 The First Affiliated Hospital, Shantou University Medical College, 515041 Shantou, China; 2 School of Public Health, Shantou University, 515041 Shantou, China; 3 Injury Prevention Research Center, Shantou University Medical College, 515041 Shantou, China

**Keywords:** Gamma-glutamyl transpeptidase to platelet ratio, Gamma-glutamyl transpeptidase to lymphocyte ratio, Reference range

## Abstract

**Background:**

Gamma-glutamyl transpeptidase to platelet ratio (GPR) and gamma-glutamyl transpeptidase to lymphocyte ratio (GLR) are assumed to be prognostic factors in liver fibrosis, cirrhosis and hepatocellular carcinoma. However, the reference values of GPR and GLR were not known.

**Objectives:**

The study aimed to investigate the reference ranges of GPR and GLR in Chinese Han population in Chaoshan region in South China.

**Methods:**

A retrospective study was conducted in the First Affiliated Hospital of Shantou University Medical College in South China. 2400 healthy adults aged 20~79 years were included. GPR and GLR were determined.

**Results:**

Of 2400 healthy adults, 1200 men and 1200 women were included. The median GPR and GLR for men were 0.22 and 11.28, for women were 0.18 and 7.86, respectively. The 95% reference range of GPR in normal male and female are 0.09~0.54 and 0.08~0.55, GLR are 4.55~29.64 and 3.52~23.08, respectively. The male had a higher GPR at age 20~49 than the female while the GPR at age 60~79 was higher in the female than in the male. The GPR was affected by age, decreased with aging in male and increased in female. The GLR was higher in the male than in the female and varied with aging in the female but not in the male.

**Conclusion:**

The study provides reference data on GPR and GLR from different age and sex groups in South China. GPR and GLR varied with age and sex.

## Introduction

Chronic hepatitis B virus (HBV) infection is a major public health problem, especially in countries of the African region and Western Pacific region, with HBsAg seroprevalence 8.83% and 5.26%, respectively [1]. It was estimated that HBsAg prevalence was 3.61% worldwide and about 248 million people were HBsAg positive in 2010 [1]. Hepatitis B is a common cause of liver fibrosis, which may lead to cirrhosis, liver failure and hepatocellular carcinoma if untreated. Therefore, it is important to identify liver fibrosis for decreasing the burden of disease attributable to HBV infection.

Recently Lemoine M et al. reported that the gamma-glutamyl transpeptidase (GGT) to platelet ratio (GPR) can predict significant liver fibrosis and cirrhosis in patients with chronic HBV infection in West Africa [2], which can be used in other populations and hepatitis B virus-related liver cancer [3–7]. More recently, gamma-glutamyl transpeptidase to lymphocyte ratio (GLR) were reported to be predictors of microvascular invasion in hepatocellular carcinoma [8] and intrahepatic cholangiocarcinoma patients following hepatectomy [9]. These tests supplied simple, cheap and convenient methods to assess the hepatic fibrosis level of patients with chronic hepatitis B and prognosis of patients with hepatocellular carcinoma.

Though there have been extensive investigations on GPR and GLR, the normal ranges of GPR and GLR were not investigated. It is important to investigate the ranges of GPR and GLR. The aim of this study is to explore the reference values of GPR and GLR among the Han population in Chaoshan District of Guangdong Province in South China.

## Methods

The study was conducted retrospectively in the First Affiliated Hospital of Shantou University Medical College in South China. Hepatic function and Complete blood count (CBC) tests between January 2019 and December 2019 were reviewed from healthy persons aged 20~79 years without diagnosed diseases including acute or chronic infection, heart failure, renal failure, autoimmune or hematopoietic diseases, acute or chronic hepatitis, hepatic fibrosis, cirrhosis and hepatocellular carcinoma. The healthy adults had no history of smoking, alcohol abuse and drug usage for diseases as noted above and were divided into groups according to gender and age. A total of 2400 healthy adults were included. Blood samples were obtained from fasting subjects. Platelet and lymphocyte cell counts were determined by the Coulter method with the Beckman Coulter LH780 analyzer. The near month-coefficient of variation of platelet and lymphocyte cell was 3.53% and 2.14%, respectively (The set value of coefficient of platelet and lymphocyte cell variation was 4.49% and 3.49%). GGT was determined by the rate method with Beckman Coulter AU5800. The near month-coefficient of GGT variation was 1.19% (The set value of coefficient of variation was 3.0*%*).

GPR and GLR were calculated as GGT/ULN of GGT/platelet count (10^9^/L)×100 [2] and as the ratio of GGT to lymphocyte cell count [8], respectively. The samples were excluded with White blood cell (WBC) less than 3.5 × 10^9^/L or more than 9.5 × 10^9^/L, lymphocyte less than 1.1 × 10^9^/L or more than 3.2 × 10^9^/L and platelet less than 125 × 10^9^/L or more than 350 × 10^9^/L and hepatic insufficiency with GGT more than 100 U/L. The study was approved by the ethics committee of Shantou University Medical College.

Data are presented as mean ± SD or median and interquartile range. Differences between group means were assessed by an unpaired Student’s t-test or Mann-Whitney test for single comparisons or by Kruskal-Wallis test for multiple comparisons using SPSS 24.0. P value < 0.05 was considered significant.

## Results

**Fig. 1:**
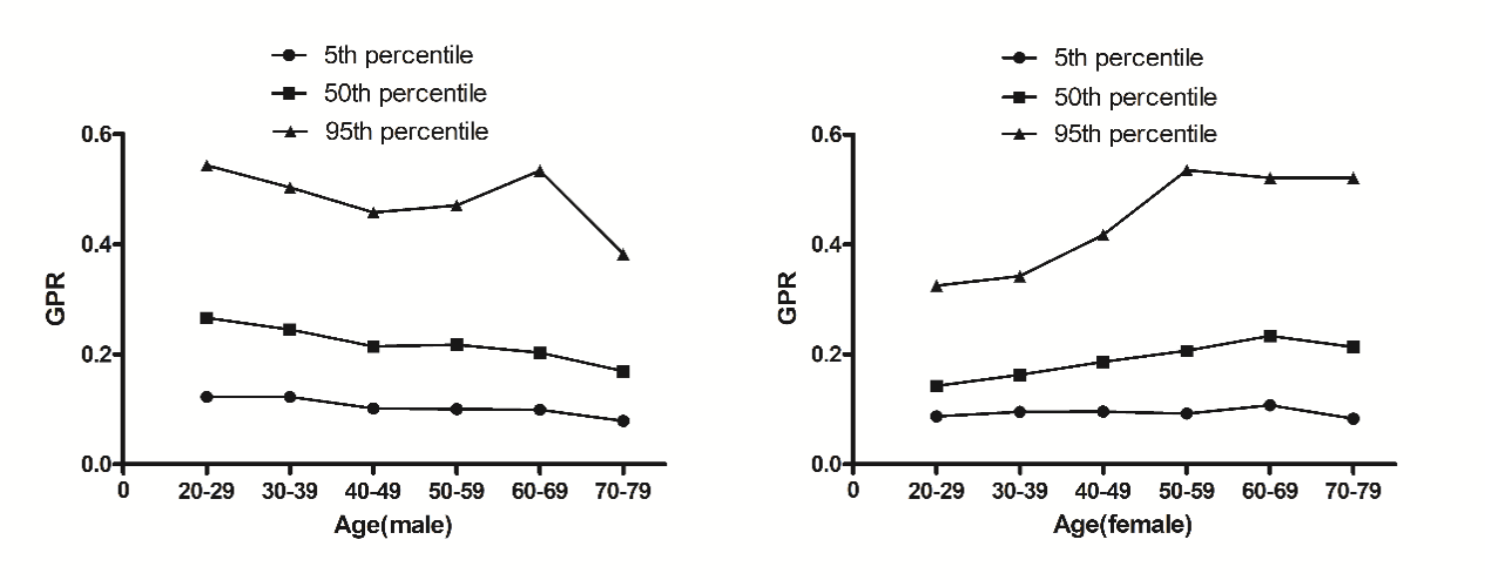
The percentile nomogram for GPR in the male and the female

**Fig. 2:**
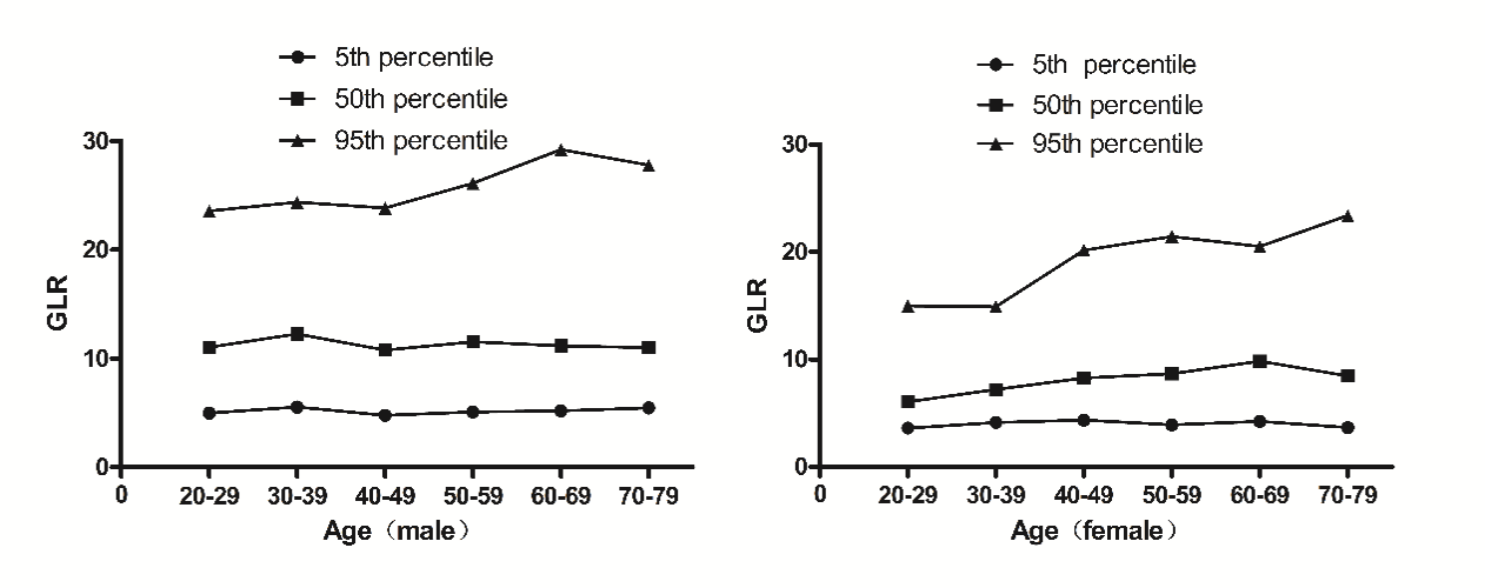
The percentile nomogram for GLR in the male and the female

As shown in the Table 1, there are 2400 individuals in present study, which included 1200 men and 1200 women. The differences of age between the male and female were not significant. The male had a higher GGT, lymphocyte cell counts, GPR and GLR than the female while the female had a higher platelet counts. The median GPR and GLR for men and women were 0.22, 11.28, 0.18 and 7.86, respectively. The 95% reference range of GPR in normal male and female are 0.09~0.54 and 0.08~0.55, GLR are 4.55~29.64 and 3.52~23.08, respectively. GPR and GLR were analyzed based on sex and age (shown in Figs. 1 and 2; Tables 2 and 3,). The GPR was affected by age, decreased with aging in male and increased in female. There are significant differences of GPR among at age 20~39, 40~69 and 70~79 in the male and among at age 20~29, 30~39, 40~49 and 50~79 in the female. The GPR did not vary from age 20 to 39, 50 to 69, respectively in the male and from age 50 to 79 in the female. The male had a higher GPR at age 20~49 than the female while the GPR at age 60~79 was higher in female than in male. At age 50~59 group there are no difference of the GPR between the male and the female. The differences of GLR between the male and female were significant different from age 20 to 79. The effect of aging on GLR varied with sex. There was significant effect of aging on GLR in the female while not in the male.

**Table 1:** Main characteristics of the study based on sex

	Male	Female	*P* Value
Number	1200	1200	
Age (years, mean ± SD)	49.40 ± 16.96	49.29 ± 16.79	0.869
Median GGT (U/L) (IQR)	26.00 (19.00–36.00)	18.00 (13.00–25.00)	0.000
Mean PLT (×10^9^/L)	205.58 ± 41.55	219.78 ± 42.15	0.000
Mean LY (×10^9^/L)	2.36 ± 0.45	2.31 ± 0.47	0.008
Median GPR (IQR)	0.22 (0.15–0.31)	0.18 (0.14–0.26)	0.000
95% reference range	0.09 ~ 0.54	0.08 ~ 0.55	
Median GLR (IQR)	11.28 (8.03–16.06)	7.86 (5.83–11.15)	0.000
95% reference range	4.55 ~ 29.64	3.52 ~ 23.08	

GGT, gamma-glutamyl transpeptidase; PLT, platelet; LY, lymphocyte; GPR, GGT to platelet ratio; GLR, GGT to lymphocyte ratio

**Table 2:** Gamma-glutamyltranspeptidase-to-platelet ratio at different groups

Subgroup (age)	Gamma-glutamyltranspeptidase-to-platelet ratio (male)	Gamma-glutamyltranspeptidase-to-platelet ratio (female)	*P* value
20 ~ 29	0.27 (0.19–0.35)	0.14 (0.11–0.18) ^∆∆^	0.000
30 ~ 39	0.24 (0.18–0.32)	0.16 (0.13–0.21)*	0.000
40 ~ 49	0.21 (0.15–0.29) ^∆^	0.19 (0.15–0.25) ^##^	0.033
50 ~ 59	0.22 (0.16–0.32) ^∆^	0.21 (0.15–0.29)	0.204
60 ~ 69	0.20 (0.14–0.28) ^∆^	0.23 (0.17–0.31)	0.002
70 ~ 79	0.17 (0.12–0.25) ^∆ #^	0.21 (0.14–0.31)	0.001

^∆^compared with group age 20 ~ 39 (*P* < 0.05) ^#^compared with group age 20 ~ 69(*P* < 0.01)

^∆∆^compared with group age 30 ~ 79(*P* < 0.05)*compared with group age 40 ~ 79 (*P* < 0.05)

^##^ compared with group age 50 ~ 79(*P* < 0.05)

**Table 3:** Gamma-glutamyltranspeptidase-to-lymphocyte ratio at different groups

Subgroup (age)	Gamma-glutamyltranspeptidase-to-lymphocyte ratio (male)	Gamma-glutamyltranspeptidase-to-lymphocyte ratio (female)	*P* value
20 ~ 29	11.03 (8.31–14.81)	6.06 (5.08–7.41)^#^	0.000
30 ~ 39	12.26 (8.03–16.58)	7.21 (5.63–9.56)^∆^	0.000
40 ~ 49	10.80 (7.55–15.60)	8.27 (6.18–11.57)	0.000
50 ~ 59	11.53 (8.25–17.46)	8.67 (6.51–12.13)	0.000
60 ~ 69	11.16 (8.22–16.83)	9.83 (6.74–13.34)	0.000
70~79	11.02 (7.98–15.95)	8.45 (5.93–12.58)	0.000

^#^compared with group age 30 ~ 79 (*P* < 0.05); ^∆^compared with group age 40 ~ 79 (*P* < 0.01)

## Discussion

Liver fibrosis is a wound-healing response to liver injury caused by various factors such as viral hepatitis, alcohol abuse, and non-alcoholic steatohepatitis (NASH) and non-alcoholic fatty liver disease (NAFLD). Though liver fibrosis is a reversible process, advanced liver fibrosis can result in cirrhosis, liver failure and hepatocellular carcinoma (HCC). It is important to assess fibrosis stage. Liver biopsy has been considered the gold standard for diagnosing liver fibrosis, however it is invasive and can cause serious complications [10, 11]. Transient elastography is a noninvasive tool for staging liver fibrosis, but the Fibroscan device is expensive. Furthermore the diagnostic accuracy of the method was poorer for significant fibrosis [12]. Recently GPR and GLR were reported to be predictors of liver fibrosis, cirrhosis and hepatocellular carcinoma [2–9]. However, it has remained unknown about the reference ranges of GPR and GLR in healthy adults.

In present study we measured the GPR and GLR in 2400 Chinese healthy adults. We found that the 95% reference range of GPR in normal male and female are 0.09~0.54 and 0.08~0.55, GLR are 4.55~29.64 and 3.52~23.08, respectively. The GPR and GLR were affected by sex and age.

GGT, a cell-membrane-bound protease, has long been regarded as a marker of liver disease [13]. Recent evidences have shown the association between GGT and cancer [14, 15], cardiovascular diseases, lung inflammation and neurological diseases [16]. Moreover it has been found that GPR and GLR are associated with significant liver fibrosis, cirrhosis and liver cancer [2–9]. There was significant positive correlation between GPR and fibrosis stage. The optimal cut-off value of GPR for significant fibrosis and cirrhosis was 0.32 and 0.56, respectively [2]. High GPR (> 0.23) was an independent risk factor for hepatocellular carcinoma development in chronic hepatitis patients [17]. High GLR was also an independent prognostic factor of hepatocellular carcinoma and intrahepatic cholangiocarcinoma [8, 9].

It has been demonstrated that there are significant male-female differences in the reference range for serum or plasma GGT [18], platelet and lymphocyte cell counts [19]. Furthermore, the geographic and ethnic difference of platelet counts was significant [20–22]. These studies showed that GPR and GLR varied significantly among sex, geographic region and race.

Though GPR and GLR were used widely in many diseases, the cut-off points for risk stratification varied in these studies, which were affected by the disease category, age, and race of patients. In the studies from West Africa a lower cut-off value of GPR for predicting significant liver fibrosis was suggested than that in China (0.32 vs. 0.448). The optimal cut-off value of GLR was 33.7 for predicting prognosis of intrahepatic cholangiocarcinoma while 56 for hepatocellular carcinoma [8, 9]. In present study, we found that GPR and GLR varied with age and sex, which suggested that factors affecting GPR and GLR should be considered when the cut-off values for risk stratification were determined.

There are a few limitations in present study. First, the study is a retrospective study and routine blood analyses were collected from healthy population in the checkup center of hospital, the effects of high alcohol consumption and use of enzyme-inducing drugs on GGT can not be excluded [18]. Secondly, owing to the geographic and ethnic difference of platelet counts [20–22], the reference range of GPR in healthy population from Han population in Chaoshan region may be different from other races in local region or other regions in China.

In summary, we found that the reference ranges of GPR and GLR in male were different from in female from Chaoshan region in South China. The GPR and GLR varied with age and sex.

## Data Availability

Raw data supporting the obtained results are available at the corresponding author.

## List of abbreviations

HBV: Hepatitis B virus
GGT: Gamma-glutamyl transpeptidase
GPR: Gamma-glutamyl transpeptidase to platelet ratio
GLR: Gamma-glutamyl transpeptidase to lymphocyte ratio
CBC: Complete blood count
WBC: White blood cell
